# How well is the female population represented in clinical trials with infusion therapies for Parkinson's disease? A systematic review and metanalysis

**DOI:** 10.1111/ene.70024

**Published:** 2025-01-05

**Authors:** Katarzyna Smilowska, Vanessa Carvalho, Natalia Szejko, João Costa, Elena Moro, Angelo Antonini

**Affiliations:** ^1^ Department of Neurology Regional Hospital Sosnowiec Poland; ^2^ Department of Neurology Unidade Local de Saúde de Santa Maria Lisbon Portugal; ^3^ Department of Clinical Neurosciences University of Calgary Calgary Alberta Canada; ^4^ Department of Bioethics Medical University of Warsaw Warsaw Poland; ^5^ Clinic of Psychiatry, Social Psychiatry and Psychotherapy Hannover Medical School Hannover Germany; ^6^ Faculdade de Medicina da Universidade de Lisboa Lisbon Portugal; ^7^ Grenoble Alpes University Division of Neurology, CHU of Grenoble, Grenoble Institute of Neurosciences Grenoble France; ^8^ Parkinson and Movement Disorders Unit, Study Center for Neurodegenerative diseases (CESNE), Department of Neuroscience, Padua Neuroscience Center (PNC) University of Padua Padua Italy

**Keywords:** female, gender equity, infusion pumps, Parkinson's disease

## Abstract

**Background:**

Parkinson's disease (PD) is a neurodegenerative disorder affecting both sexes, but differences exist between male and female in clinical manifestations, functional impact of symptoms and hormonal influences. Therefore, representativeness of females in PD trials indirectly determines the external validity of the clinical research in this field.

**Objective:**

To estimate the representativeness of female in infusion therapy trials for advanced PD.

**Methods:**

PubMed and EMBASE databases were searched (1980 to September 2023), along with congress abstracts, to identify controlled clinical trials and large non‐controlled studies on infusion therapies in PD enrolling >100 patients. Random‐effect meta‐analysis was conducted to estimate mean pooled prevalence of females included in the studies. Subgroup analyses were conducted accordingly to study design and intervention.

**Results:**

We included 15 studies (six studies on levodopa‐carbidopa intestinal gel, six on subcutaneous levodopa, two on subcutaneous apomorphine, and one on levodopa‐carbidopa‐entacapone intestinal gel). Sex was not a randomisation stratification factor in any of these studies. Only one study explored differences in the outcome estimated according to sex. Overall, the proportion of female included was 38% (95% CI:33%–43%; *I* ^2^ = 74%), without differences between studies assessing different type of interventions (*p* = 0.72) or between study design (*p* = 0.35). In two studies, females represented the majority of included patients.

**Conclusion:**

Female with advanced PD are underrepresented in infusion therapy trials. Most trials have overlooked sex‐based biological differences that can impact clinical and functional outcomes, raising concerns about the generalizability of these findings to real‐world contexts.

## INTRODUCTION

Parkinson's disease (PD) is a progressive neurological disorder that affects both sexes [1]. While research and clinical trials have made significant strides in both understanding and treating PD, there is a growing concern about the underrepresentation of female patients in these studies [2, 3]. In terms of motor phenotype, females present more frequently with a tremor‐dominant PD type, and have overall slower disease progression [4, 5]. These observations have also been recently supported by a neuroimaging study in which rates of brain aging in male were faster than in female with PD [6]. These findings could be also influenced by the observation that males overall age faster than females, even without the burden of PD [7].

Females also have higher rates of non‐motor fluctuations [8]. Moreover, they are more prone to depression and anxiety compared to males [9, 10]. Females with PD also have a higher prevalence of constipation, pain, and sleep disturbances, all affecting quality of life and functionality [11]. Hormonal factors, including oestrogen, have been implicated in influencing the development and progression of PD in females [12]. Oestrogen has neuroprotective properties, and may exert a beneficial effect on dopaminergic neurons, which are affected in PD [12, 13]. However, the scientific evidence on the relationship between oestrogen levels and PD risk and progression is conflicting. There are also a number of sex‐specific issues in females with PD that have been, until now, poorly addressed. This concerns specifically the topic of pregnancy and PD, the influence of menstrual cycle on PD symptoms, menopause and PD, and other issues related to reproductive health [14, 15].

Another important topic are different pharmacotherapy patterns between sexes. This issue has very important implications into planification of personalized treatment. One of the determinants of differences in treatment between males and females with PD is a greater levodopa bioavailability in females. This is supported by the fact that the genes involved in the levodopa metabolism, catechol‐O‐methyltransferase (COMT) and monoamine oxidase‐B (MAO‐B), are found on chromosomes 22 and X, respectively [16]. Other genetic determinants of sex‐specific response to treatment in PD have also been found; in a study by Sampaio et al., authors found that certain MAO‐B and COMT single nucleotide polymorphisms (SNP) were related to greater predisposition to levodopa induced dyskinesias in males [17]. Additionally, females exhibit greater levodopa (LD) bioavailability compared to males, as indicated by their higher area under the curve (AUC) and maximum plasma concentration (Cmax) values [16]. Since females usually have also lower body mass index, this could also influence drug availability and, as a result, increase the risk of levodopa‐induced dyskinesias [18]. There are also differences in response to non‐motor symptoms between biological sexes with males requiring earlier and higher doses of anti‐psychotic medications [19].

Among advanced therapies, deep brain stimulation (DBS) is the only one that has been studied in relation to birth sex differences [20]. Males appear to experience a greater degree of improvement following subthalamic nucleus DBS (STN‐DBS) but also face a higher risk of developing dementia after a 10‐year follow‐up [21]. Interestingly, females report better improvement of quality of life after the DBS procedure [22, 23]. So far, there are no studies directly comparing sex differences in infusion therapies although data on long term survival are available [24].

Here, we aimed to conduct a systematic review to evaluate the representativeness of females in infusion therapy studies for advanced PD.

## METHODS

The systematic review protocol was developed using guidance from the Preferred Reporting Items for Systematic Reviews and Meta‐Analyses (PRISMA) statement [25].

The literature search on PubMed and EMBASE databases was run from 1980 until September 2023 (search queries available as supplementary material). Articles in languages other than English were excluded. Abstracts presented at the European Academy of Neurology and Movement Disorders Society Congress from the past 5 years (from 2018 to 2023) were also reviewed for relevant unpublished studies. The search and study selection were conducted by three independent researchers (KS, NS, and VC).

### Inclusion criteria

We included both randomized (RCTs) and non‐randomized controlled clinical trials, as well as prospective non‐controlled studies/extensions with a sample size of at least 100 patients, that evaluated adult patients with a diagnosis of advanced PD treated with infusion therapies (subcutaneous apomorphine [CAI], levodopa‐entacapone‐carbidopa intestinal gel [LECIG], levodopa‐carbidopa intestinal gel [LCIG], and subcutaneous levodopa).

### Exclusion criteria

Reviews, case reports, case series, and observational retrospective studies, as well as *post‐hoc* analysis, were excluded. Studies evaluating non‐infusion therapies in people with advanced PD, as well as pharmacokinetic studies conducted in healthy volunteers were also excluded.

### Measured outcomes

The main outcome was the proportion of female patients enrolled in the study. Although sex and gender are not synonyms [26], we extract data interchangeable as reported in the study to defined the “female sex” variable [27].

We also extracted data on whether trials considered sex as a stratifying factor and whether studies have addressed the outcomes accordingly to the sex of the included population.

### Data collection and analysis

Data were extracted independently by three reviewers (KS, NS, and VC) using predefined forms. Disagreements were solved by consensus‐based discussion. We did not perform a formal risk of bias assessment since the certainty in studies outcome results do not have an impact in our main objective.

### Statistical analysis

Data from individual studies was pooled using STATA software 18.0 and random‐effect meta‐analyses were performed weighted by the inverse variance to estimate pooled proportion of females and 95% confidence interval (95% CI). Heterogeneity was assessed through *I* ^2^ statistics, which measures the percentage of total variation between studies attributed to interstudy heterogeneity rather than random heterogeneity [28].

Subgroup analyses were performed according to the type of infusion therapy and study type design (controlled versus non‐controlled studies).

## RESULTS

### Study selection

**FIGURE 1:**
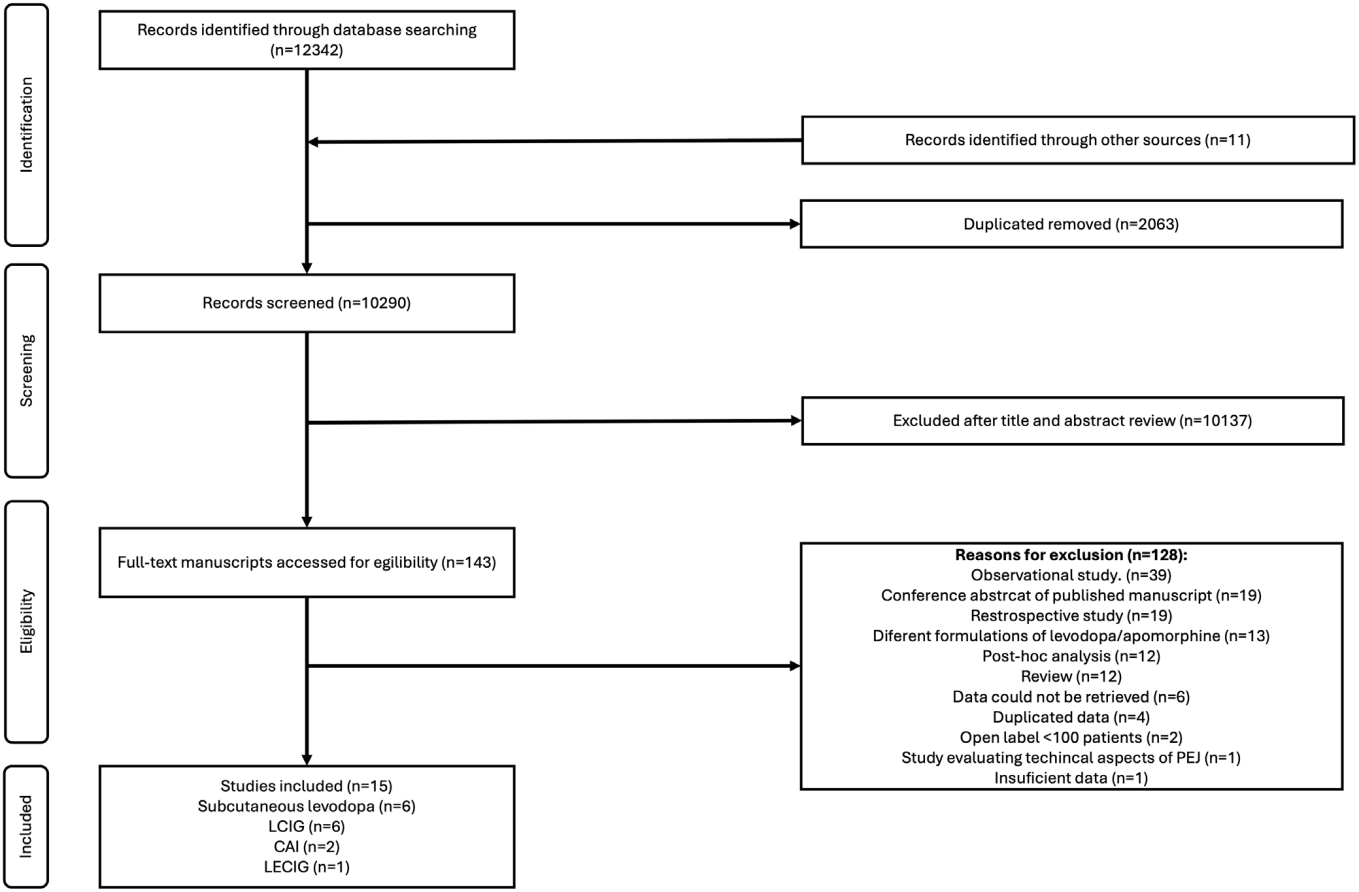
Search queries flowchart. CAI, Subcutaneous apomorphine; LCIG, Levodopa‐carbidopa intestinal gel; LECIG, Levodopa‐entacapone‐carbidopa intestinal gel; PEJ, Percutaneous endoscopic jejunostomy.

The search yielded a total of 10,289 records. After title and abstract screening, 143 studies were selected for full‐text assessment, of which 128 were rejected, and 15 fulfilled our inclusion criteria (Figure 1).

### Study characteristics

The main studies characteristics are reported in Table 1.

**TABLE 1:** Studies' characteristics.

Study	Type of study	Active group	Comparator	Outcome results by sex	Number of participants included, absolute number	Females included, absolute number (%)
Katzenschlager et al., 2018	RCT, double blind	Apomorphine subcutaneous infusion	Placebo	Yes	106	40 (37.7)
De Cock et al., 2022	RCT, double blind	Apomorphine subcutaneous infusion	Placebo	No	43	19 (44.1)
Senek et al., 2017	RCT, open label	LECIG	LCIG	No	9	4 (44.4%)
Nyhom et al., 2003	RCT, open label	LCIG	LC‐CR	No	12	2 (16.7)
Nyhom et al., 2005	RCT, open label	LCIG	OMT (oral and subcutaneous therapy)	No	25	6 (24)
Olanow et al., 2014	RCT, double blind	LCIG	LC‐IR + placebo	No	71	25 (35.2)
Fernandez et al., 2015	Non‐controlled open label study	LCIG	No comparator	No	272	152 (55.8)
Kulisevsky et al., 2020	RCT, double blind	LCIG	LC‐IR + placebo	No	13	6 (46.1)
Freire‐Alvarez et al., 2021	RCT, open label	LCIG	OMT	No	61	32 (52.4)
Giladi et al., 2021	RCT, double‐blinded	Subcutaneous levodopa/carbidopa	Placebo	No	30	9 (30)
Poewe et al., 2021	RCT, open label	24 h‐infusion subcutaneous levodopa/carbidopa	16 h‐infusion subcutaneous levodopa/carbidopa	No	214	72 (33.6)
Rosebraugh et al., 2021	Non‐controlled, non‐randomized single‐blinded	Subcutaneous levodopa/carbidopa	No comparator	No	15	4 (26.7)
Soileu et al., 2022	RCT, double‐blinded	Subcutaneous levodopa/carbidopa	LC‐IR	No	141	42 (29.7)
Aldred et al., 2023	Non‐controlled, open label study	Subcutaneous levodopa/carbidopa	No comparator	No	244	98 (40.1)
Espay et al., 2024	RCT, double blinded	Subcutaneous levodopa/carbidopa	LC‐IR + placebo	No	381	146 (38)

Abbreviations: LCIG, levodopa‐carbidopa intestinal gel; LC‐IR, levodopa/carbidopa immediate release; LECIG, levodopa‐carbidopa‐entacapone intestinal gel; OMT, optimized medical treatment; RCT, randomized clinical trial.

Overall, the 15 studies enrolled a total of 1637 patients. Twelve out of these 15 studies where RCTs (which included 67.5% of the total patients), while the remain three studies were non‐controlled studies. Six studies evaluated subcutaneous levodopa (*n* = 1025 patients overall) [29, 30, 31, 32, 33, 34], six evaluated LCIG (*n* = 454) [35, 36, 37, 38, 39, 40], two evaluated CAI (*n* = 149) [41, 42], and one evaluated LECIG (*n* = 9) [43].

The proportion of females across studies varied between 17% and 56%, with only two studies including a majority of females (52.4% and 55.8%) [38, 40]. Only of the RCTs explored differences between sexes on the outcome results [42].

### Female representativeness

**FIGURE 2:**
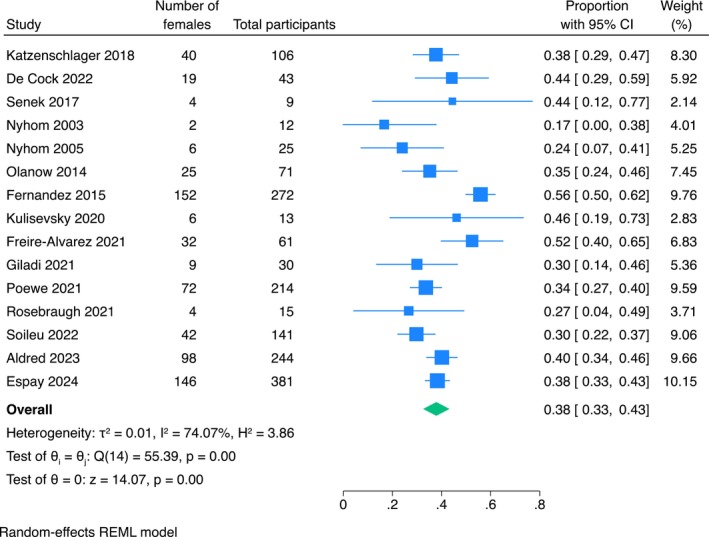
Forest plot on the proportion of females included in all the studies.

**FIGURE 3:**
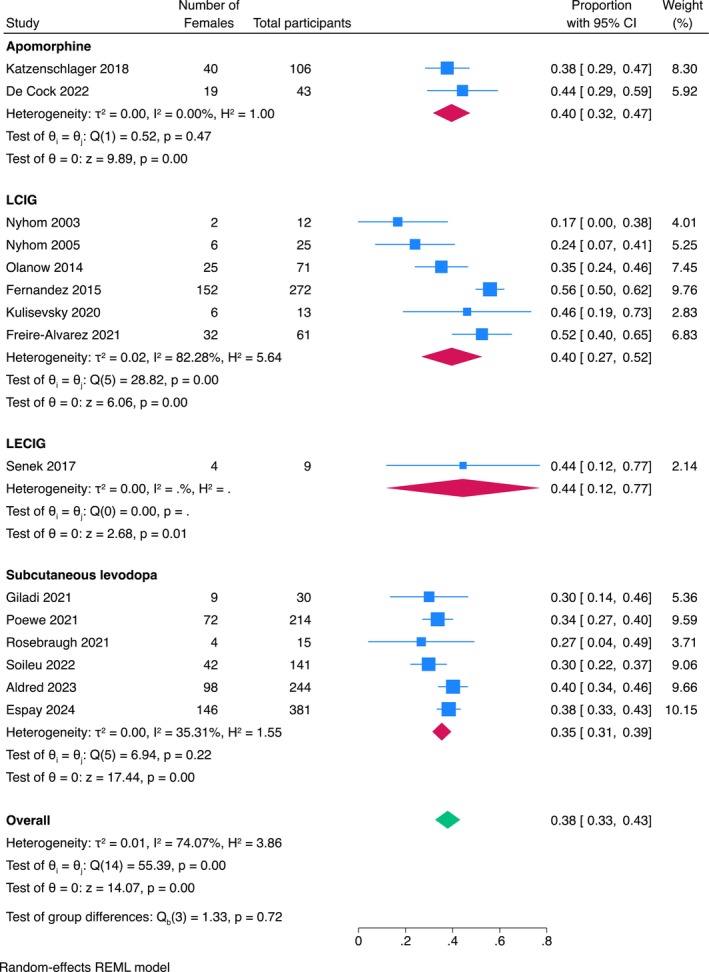
Subgroup analysis of the proportion of females included in the different treatments considered.

**FIGURE 4:**
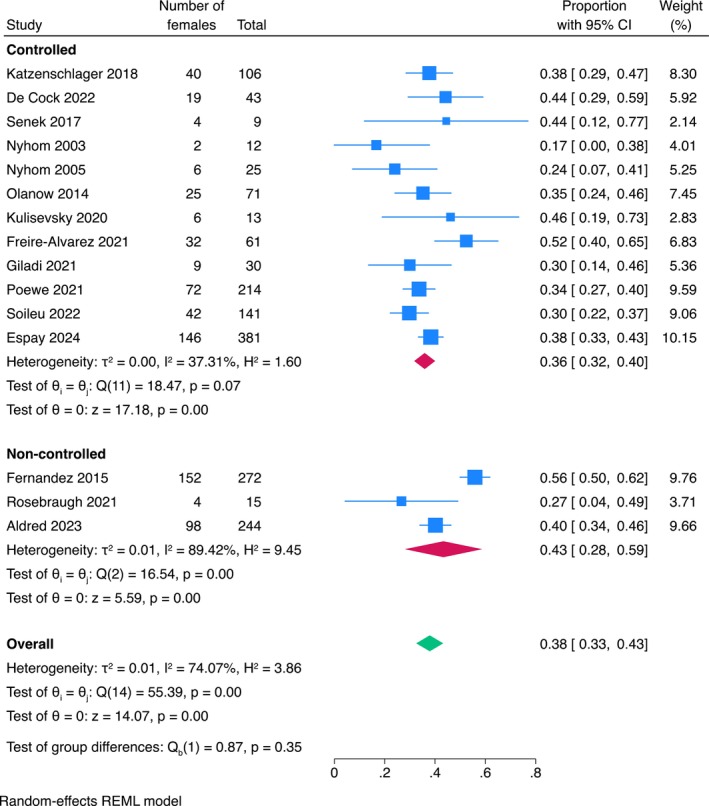
Subgroup analysis of the proportion of females included in the different study designs.

The pooled proportion of females included in studies evaluating infusion therapies for people with advanced PD was 38% (95% CI: 33%–43%; *I* ^2^ = 74%; Figure 2). There was no strong evidence that this female representativeness was significantly different between studies evaluating different infusion therapies (*p* = 0.70; Figure 3) or between studies with different designs (controlled versus non‐controlled studies) (*p* = 0.35; Figure 4).

## DISCUSSION

To the best of our knowledge, this is the first systematic review on female sex representativeness in infusion therapies in people with PD. In general, although a growing number of females is included in clinical trials, especially in RCTs, the number is still not balanced between man and females (usually 3:7). Importantly, very few studies report about subgroup analysis of efficacy depending on biological sex, although we recognise that data from subgroup analysis have important limitations [44]. When reported, none of the studies described sex differences among participants.

The inclusion of females in clinical trials has been a longstanding and critical issue, with two primary concerns [45]. Firstly, there's the recognition of potential sex‐based differences in how treatments affect individuals, underscoring the need for diverse representation in trials. Secondly, particularly in females of childbearing age, there's a valid concern about the risk of exposing foetuses to investigational drugs. Many disorders exhibit varying treatment responses between males and females, though the full extent of these differences remains elusive [46]. While some studies may lack the sample sizes necessary to discern moderate effects within subgroups, existing literature consistently advocates for analysing treatment outcomes separately in both males and females.

Overall, we found a 1.49:1 male‐to‐female ratio (ranging from 1.03:1 in LCIG to 1.76:1 in subcutaneous levodopa). The latest systematic review of PD prevalence 1.18:1 male‐to‐female prevalence ratio [47], which means that even considering the overall higher frequency of PD in males, females are still underrepresented in most trials. Therefore, there could be determinants, other than prevalence, of this difference, such as cultural, ethnical, related to healthcare access or differences in healthcare approaches among medical professionals across the globe [48, 49]. Furthermore, patient's preferences could be considered to explain at least part of this difference, as females often prefer and are more frequently treated with less invasive or aggressive treatment options in comparison to males due to differences in patient's values and preferences [50, 51]. However, we found a lower male to female ratio in more invasive techniques such as LCIG (1.04:1) and LECIG (1.25:1) than subcutaneous levodopa (1.76:1) and apomorphine (1.52:1).

Conversely, Meinert and colleagues, performed a systematic review in mid‐1990's and argued there was little evidence to support the notion that females were underrepresented in trials [52]. The authors reported 65.3% trials including males and females, 10.1% involving only males and 10.7% involving only females. There was, however, a tendency for smaller trials to be male only and, although, for instance, in trials for heart disease, 64% involved both male and female patients, 13.9% of trials included only males and 0.08% only females. Regarding the prevalence of females included in the trials that recruited both sexes, this seemed to vary according to the disease, and while in oncology females outnumbered males in 1.55 to 1, in cardiology, males outnumbered females in 3.66 to 1 [52].

A systematic review of representation of females with PD in RCTS found a similar asymmetry. Tosserams and colleagues reported that, when evaluating trials published since 2010, the majority (55.7%), recruited over 59% of males [45]. In our study, the percentage of females included in the studies oscillated between 30%–40%. This is similar to reports from studies about other advanced therapies in PD. In a recently published study from Germany, the proportion of females enrolled for DBS study in PD was, similarly, 30% [53]. While both males and females profited from the procedure when it comes to motor performance, only females improved in general cognition, while men improved in terms of depressive symptoms and impulsivity. Likewise, the inclusion of female patients in infusion therapies in PD is lower than the one found, for instance, in Alzheimer's disease—where 59% of the participants recruited were female [54, 55]. However, even in this case, if we consider that 62%–68% of the patients with Alzheimer's disease are females, this value Is higher than the proportion of females included in clinical trials [54].

### Future directions

As we consider future approaches to addressing this disparity, we have identified five key strategies worth exploring.

#### Sex‐specific research and clinical trials

Future research efforts should prioritize the inclusion of an adequate representation of females. This involves designing studies with balanced sex ratios and conducting subgroup analyses to assess treatment efficacy, safety, and tolerability specifically in female participants. Moreover, exploring potential sex‐based differences in disease progression, symptomatology, and treatment response can provide valuable insights into tailoring infusion therapies to meet the unique needs of females with PD.

#### Understanding biological mechanisms

Investigating the biological mechanisms underlying sex‐based differences in PD pathophysiology and treatment response is crucial. This includes exploring the influence of sex hormones (both endogenous and exogenous), genetic factors, and neurobiological pathways on disease progression and therapeutic outcomes. Identifying biomarkers that predict treatment response in females can guide personalized treatment approaches and enhance clinical decision‐making.

#### Addressing socioeconomic and cultural factors

Socioeconomic and cultural factors may contribute to the underrepresentation of female in infusion therapies for PD. Future research should examine barriers to access, including disparities in healthcare access, financial constraints, and cultural beliefs that may disproportionately affect females. Implementing targeted interventions, such as educational programs, financial assistance, and community outreach initiatives, can help address these barriers and promote equitable access to infusion therapies for all individuals with PD.

#### Developing sex‐sensitive treatment guidelines

Developing sex‐sensitive treatment guidelines for PD infusions and device aided therapies in general can help healthcare providers optimize treatment management for female patients. This involves tailoring treatment regimens, dosing strategies, and monitoring protocols to account for sex‐specific differences in disease presentation and response to therapy. Collaboration among healthcare professionals, patient advocacy groups, and regulatory agencies is essential to develop and implement these guidelines effectively.

#### Promoting gender diversity in PD research

Efforts to promote sex diversity in PD research, including increasing the representation of female and gender‐diverse investigators and research participants, are essential. Funding agencies, academic institutions, and research organizations can support initiatives aimed at enhancing diversity and inclusion in PD research through targeted funding opportunities, mentorship programs, and training initiatives. By fostering sex and gender diversity in research, we can improve our understanding of sex and gender‐based differences in PD and advance the development of personalized treatment strategies.

## CONCLUSION

Females continue to be underrepresented in PD clinical trials including those for infusion therapies. Effectively addressing this underrepresentation necessitates a holistic approach spanning research, clinical practice, and policy development. This can be achieved by prioritizing research methodologies that account for sex differences, fostering inclusivity in clinical trial recruitment, and tackling socioeconomic and cultural barriers to access. By implementing these measures, we can strive towards achieving equitable and optimal treatment outcomes for all individuals affected by PD, irrespective of their sex or gender.

## AUTHOR CONTRIBUTIONS

**Katarzyna Smilowska:** Conceptualization; writing – original draft; methodology; formal analysis; data curation; resources; investigation. **Vanessa Carvalho:** Conceptualization; investigation; writing – original draft; methodology; software; formal analysis; data curation; resources. **Natalia Szejko:** Conceptualization; writing – review and editing; methodology; data curation. **João Costa:** Conceptualization; writing – review and editing; methodology; formal analysis; supervision. **Elena Moro:** Writing – review and editing; supervision; conceptualization. **Angelo Antonini:** Conceptualization; methodology; validation; writing – review and editing; supervision.

## FUNDING INFORMATION

Katarzyna Smilowska: The author declare that there are no additional disclosures to report. Vanessa Carvalho: The author declares that there are no additional disclosures to report. Natalia Szejko: received funding from the Medical University of Warsaw, Polish Ministry of Heath, American Academy of Neurology, American Brain Foundation and Tourette Association of America. João Costa: The author declares that there are no additional disclosures to report. Angelo Antonini: received compensation for consultancy and speaker‐related activities from UCB, ConvaTec, Infusion Care, Bayer, General Electric, Britannia, AbbVie, Zambon, Bial, Theravance Biopharma, TreeFrog Therapeutics, Roche, and Medscape. He receives research support from Bial, Lundbeck, Roche, Angelini Pharmaceuticals, Horizon 2020 Grant 825,785, Horizon 2020 Grant 101,016,902, Ministry of Education University and Research (MIUR) Grant ARS01_01081, Cariparo Foundation, and Movement Disorders Society for NMS Scale Validation. Elena Moro: has received honoraria from Medtronic for consultant service; she also received research grant support from Abbott, Grenoble Alpes University, and France Parkinson.

## CONFLICT OF INTEREST STATEMENT

The authors declare that there are no conflicts of interest relevant to this work.

## ETHICS STATEMENT

Informed patient consent was not necessary for this work. We confirm that we have read the Journal's position on issues involved in ethical publication and affirm that this work is consistent with those guidelines.

## Data Availability

The data that support the findings of this study are available from the corresponding author upon reasonable request.
